# Efficient Screening of Marine Extracts for Protease Inhibitors by Combining FRET Based Activity Assays and Surface Plasmon Resonance Spectroscopy Based Binding Assays

**DOI:** 10.3390/md11114279

**Published:** 2013-10-30

**Authors:** Tony Christopeit, Kersti Øverbø, U. Helena Danielson, Inge W. Nilsen

**Affiliations:** 1 Nofima AS, Muninbakken 9-13, Tromsø 9291, Norway; E-Mails: kersti.overbo@nofima.no (K.Ø.); inge-w.nilsen@nofima.no (I.W.N.); 2 Department of Chemistry—BMC, Uppsala University, Box 576, Uppsala 751 23, Sweden; E-Mail: helena.danielson@kemi.uu.se

**Keywords:** HIV-1 protease, secreted aspartic proteases, marine vertebrates, Norwegian spring spawning herring, *Clupea harengus* L.

## Abstract

The screening of extracts from marine organisms is a widely used strategy to discover new drug leads. A common problem in the screening process is the generation of false positive hits through unspecific effects from the complex chemical composition of the crude extracts. In this study, we explored a combination of a fluorescence resonance energy transfer (FRET) based activity assay and a surface plasmon resonance (SPR) based binding assay to avoid this problem. An aqueous extract was prepared from rest raw material of the Norwegian spring spawning herring, and further fractionated by methanol solubility and solid phase extraction. FRET based activity assays were used to determine the influence of each extract on the activity of different proteases. Several extracts showed more than 50% inhibition. The inhibition mechanisms were elucidated by SPR based competition experiments with known inhibitors. For the secreted aspartic proteases 1, 2, 3 and HIV-1 protease, the results indicated that some extracts contain inhibitors interacting specifically with the active site of the enzymes. The study shows that a combination of an activity assay and an SPR based binding assay is a powerful tool to identify potent inhibitors in marine extracts. Furthermore, the study shows that marine vertebrates offer an interesting source for new bioactive compounds, although they have rarely been explored for this purpose.

## 1. Introduction

Small organic molecules produced by marine organisms are a vast source for novel bioactive compounds and drugs leads [1]. During the last decades, new bioactive compounds with anti-cancer, anti-bacterial and anti-fungal activity have been isolated from marine sources, proving the high potential of marine drug discovery [2,3].

One of the first steps in marine drug discovery is the production of crude fractionated extracts from a selected marine source [4]. Extracts containing bioactive compounds are identified by different types of screening assays. In phenotypic based cell assays, the presence of bioactive compounds is indicated by the influence on the proliferation or viability of e.g., cancer cells or pathogenic microorganism. Target based cell assays utilize genetically modified cells expressing a drug target coupled to a reporter system. In contrast, cell free assays use pure proteins to measure the influence on a special drug target [5,6]. However, a problem with all these assays is the generation of false positive hits, especially during screening of crude marine extracts with their complex chemical compositions [7].

A widely used type of screening assay to identify bioactive compounds inhibiting proteases, an important class of drug targets, are fluorescence resonance energy transfer (FRET) based activity assays due to the simple design of substrates, the high sensitivity of the read out and the real time monitoring of cleavage [8]. FRET based activity assays give direct information about the inhibitory effects of an extract. However, only little information is obtained about the inhibition mechanism. Hence false positives are often found, caused by the complex chemical composition of the extracts influencing the assay, e.g., interaction with the substrate, changes in pH or influence on the fluorescence read out. A more recently developed type of screening assay to study protease inhibitors involves the analysis of binding to the target, using surface plasmon resonance spectroscopy (SPR) [9,10,11]. Such assays enable the elucidation of the interaction mechanism and the discrimination between specific and unspecific interactions. In this way, SPR based binding assays allow the identification of false positive hits from activity assays and are hence a good complement. However, SPR based binding assays give no information about the inhibitory effects of an extract, which makes the combination with activity assays inevitable. Despite the clear advantages of the method and the widely use for the screening of chemical libraries [12], SPR rarely has been applied to extracts from natural sources [13].

The process of marine drug discovery is strongly dependent on the supply of sufficient biological material of the marine source for identification, isolation and structure determination of a bioactive compound. However, the marine invertebrates and microorganisms used in marine drug discovery are often only available in small quantities, expensive to collect, or in the, case of microorganism, difficult to cultivate [14,15]. On the other hand, marine vertebrates are available in large amounts, often as rest material from the fishing industry. Furthermore, these large amounts of biological material often have a constant composition due to the collection under similar conditions. Despite these clear advantages, marine vertebrates have rarely been used in marine drug discovery [1].

Proteases are important drug targets for many different diseases and several protease inhibitors are now in clinical use, targeting, e.g., HIV-1 protease, renin and thrombin [16]. Furthermore, several proteases are currently under investigation as promising drug targets, like secreted aspartic proteases (SAP) for candidiasis [17], the human cytomegalovirus (HCMV) protease for HCMV [18] and the β-site amyloid precursor protein cleaving enzyme 1 (BACE1) for Alzheimer’s disease [19].

In this study, we explored extracts from the Norwegian spring spawning herring for inhibitors of the proteases SAP1, 2 and 3 from *Candida albicans*, HIV-1 protease, pepsin, BACE1 and HCMV protease. A novel approach was used by combining a FRET based activity assay and an SPR based binding assay. The FRET based activity assay allowed the identification of extracts inhibiting the proteases, whereas the SPR based binding assay elucidated the mechanism causing the inhibition. In this way it was possible to identify extracts containing promising protease inhibitors.

## 2. Results and Discussion

**Figure 1:**
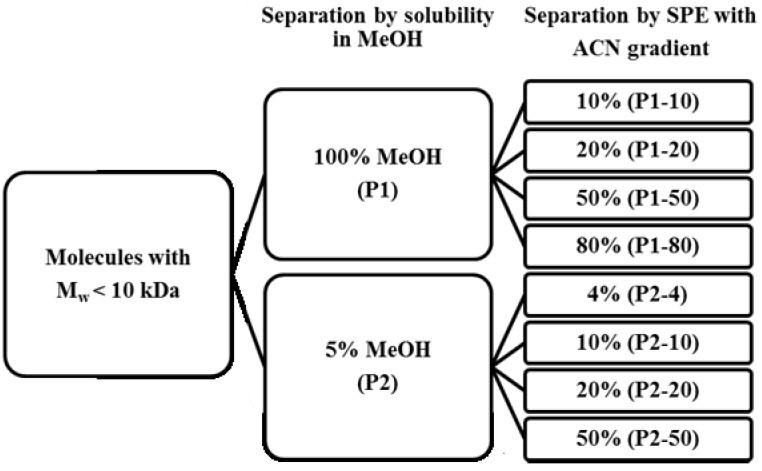
Separation scheme for the crude extracts using differential solubility in MeOH and solid-phase extraction (SPE). Soluble material was first extracted with 100% and 5% MeOH. For further fractionation by SPE, the extracts were loaded onto a C18 column and eluted with different acetonitrile (ACN) concentrations. The nomenclature for the extracts is shown in brackets.

An extract containing low molecular weight compounds (MW < 10 kDa) was prepared from rest raw material of the Norwegian spring spawning herring. The extract was further fractionated by differential solubility in methanol and solid-phase extraction (SPE), using a C18 column and an acetonitrile (ACN) gradient (Figure 1). The resulting extracts were screened for protease inhibition by FRET based activity assays. In addition, extracts were subsequently screened by an SPR based binding assay to verify true inhibitors or to discharge false positive hits.

### 2.1. Screening for Inhibitors of HIV-1 Protease, SAP1, SAP2, SAP3 and Pepsin

HIV-1 protease, SAP1, 2 and 3 from *Candida albicans* and pepsin belong to the group of aspartic proteases and share a common catalytic mechanism. Despite their different origin from a vertebrate, a fungus and a retrovirus, their active sites have high structural similarities and interact with the same active site inhibitors, e.g., acetyl-pepstatin and saquinavir [10,20,21]. The results from the FRET based activity assay and the SPR based binding assay were similar for HIV-1 protease, SAP1, SAP2, SAP3 and pepsin.

In the FRET based activity assay, all extracts were screened for protease inhibition in a dilution of 1:300 (Table 1). The dilution was to be chosen as low as possible to ensure the detection of low inhibitor amounts in the extracts. However, dilutions lower than 1:300 resulted in strong background signals, interfering with the read out of the FRET based activity assay.

**Table 1:** Inhibition of protease activities by extracts from *Clupea harengus*. Inhibition higher than 50% is highlighted (bold). Errors were calculated as the standard deviation from three independent experiments.

	% Inhibition						
Extract	HIV-1 protease	SAP1	SAP2	SAP3	Pepsin	BACE1	HCMV Protease
P1-10	27 ± 1	11 ± 1	−5 ± 6	−6 ± 1	5 ± 2	7 ± 1	41 ± 4
P1-20	**70** ± 13	47 ± 1	36 ± 15	44 ± 1	34 ± 2	44 ± 3	**71** ± 4
P1-50	**56** ± 6	**75** ± 11	**68** ± 4	**76** ± 3	47 ± 13	27 ± 3	**68** ± 10
P1-80	−1 ± 1	29 ± 4	**60** ± 1	**51** ± 1	**54** ± 14	2 ± 4	45 ± 5
P2-4	11 ± 1	10 ± 7	4 ± 11	6 ± 4	11 ± 11	3 ± 3	43 ± 6
P2-10	14 ± 3	21 ± 8	−5 ± 4	8 ± 4	10 ± 5	11 ± 3	49 ± 2
P2-20	28 ± 3	−5 ± 15	7 ± 1	−2 ± 7	12 ± 1	22 ± 4	30 ± 9
P2-50	−18 ± 4	8 ± 5	36 ± 13	14 ± 1	13 ± 6	9 ± 1	10 ± 3

Extracts P1-20 and P1-50 reduced the protease activities by more than 30% and 45%, respectively. Extract P1-80 inhibited all proteases, except HIV-1 protease, by more than 30%. Extract P2-50 increased the activity of the HIV-1 protease. All other extracts had only weak effects on the protease activities. For confirmation of the results obtained with the 1:300 dilutions, all extracts were also tested at a dilution of 1:600. The results from both dilutions were in accordance, although inhibition was higher with the lower dilution 1:300. The mechanisms causing the detected inhibitions were not clear and hence an SPR based binding assay was used to elucidate the inhibition mechanism.

In the SPR based binding assay, all extracts were analyzed using an active surface with the immobilized protease and an empty surface for reference corrections. Several extracts produced sensorgrams with concentration dependent signals (data not shown). However, the interpretation of the sensorgrams was difficult due to high bulk effects, a common problem in SPR spectroscopy, especially for complex samples or if there are large differences between the active and the reference surfaces [22]. Additionally, the steady state plots showed a linear concentration dependency and high saturation values, typical for nonspecific binding which can mask specific interactions [23]. To overcome these problems alternative experimental setups for the SPR based binding assay were developed.

**Figure 2:**
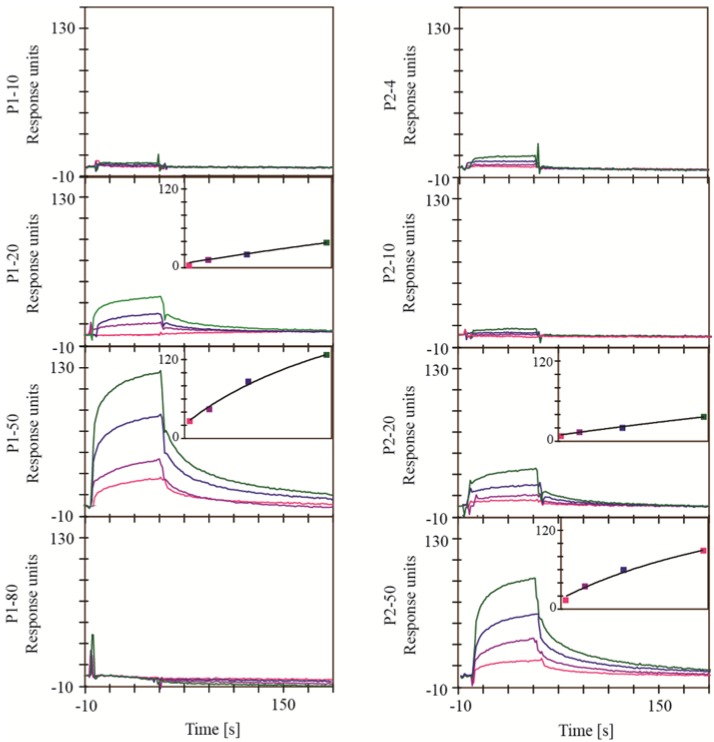
Sensorgrams from the surface plasmon resonance (SPR) based binding assay for the interaction of the extract with HIV-1 protease using experimental setup A. A surface with immobilized HIV-1 protease and the active site blocked by saquinavir was used for reference correction. Extracts were analyzed in dilutions of 1:80 (green), 1:160 (blue), 1:320 (purple) and 1:640 (pink). Responses are shown as absolute responses. Insets show the steady state plots.

In the experimental setup A, a surface with the immobilized protease and the active site blocked by an inhibitor was used for reference correction. Since the only difference between the active and the reference surface was the blocking of the active site, it was expected to reduce signals from bulk effects and nonspecific interactions. Furthermore, this experimental setup allowed identification of extracts containing compounds, which compete with inhibitors binding to the active site of a protease. However, this type of experimental setup is dependent on the availability of an active site inhibitor with a slow dissociation. For the HIV-1 protease, the active site inhibitor saquinavir meets this requirement and was hence used to prepare the reference surface [24]. Every extract was analyzed at four different concentrations (Figure 2).

Extracts P1-20, P1-50, P2-20 and P2-50 showed sensorgrams with association and dissociation phases indicative of actual interactions. The corresponding steady state plots showed concentration dependency and saturations levels between 230 and 300 RU, reasonable for an interaction with a small molecule. Hence, it can be assumed that the extracts contain compounds specifically interacting with the active site of the HIV-1 protease. For SAP1, SAP2 and SAP3, an inhibitor with sufficiently slow dissociation was not available for preparation of a stable reference surface. Experimental setup B was therefore developed to test the extracts.

**Figure 3:**
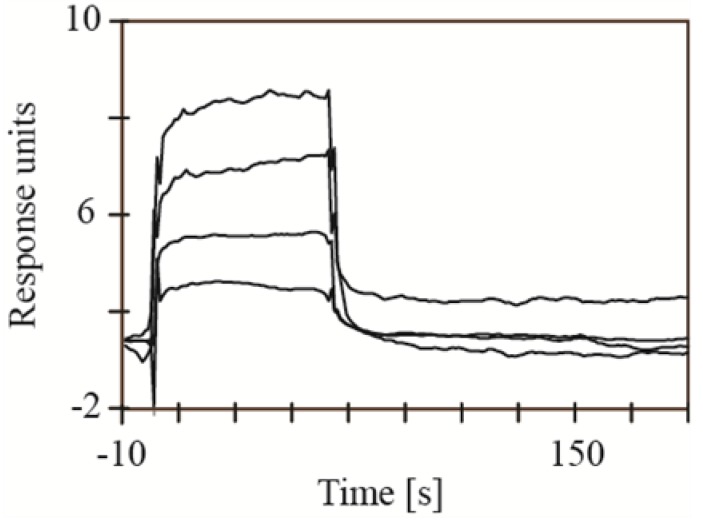
Interaction of acetyl-pepstatin with HIV-1 protease using experimental setup B. Acetyl-pepstatin was analyzed using 10, 20, 40 µM and 80 µM. Sensorgrams recorded in the presence of saquinavir were used for reference correction.

In the experimental setup B, every extract was analyzed in the presence and the absence of an active site inhibitor. The sensorgrams obtained in the presence of the active site inhibitor were used for reference correction. In this way, it was possible to remove signals from nonspecific binding as well as bulk effects. To validate this type of experimental setup, it was used to study the interaction between HIV-1 protease and acetyl-pepstatin (Figure 3). Although the quality of the obtained sensorgrams were not good enough to determine kinetic values, probably due to secondary effects caused by the competition of the inhibitors, it was clearly possible to detect an interaction. Furthermore, the sensorgrams indicate an affinity in a µM range for acetyl-pepstatin, which is in accordance with the literature [9]. Hence, experimental setup B is suitable to study the marine extracts.

**Figure 4:**
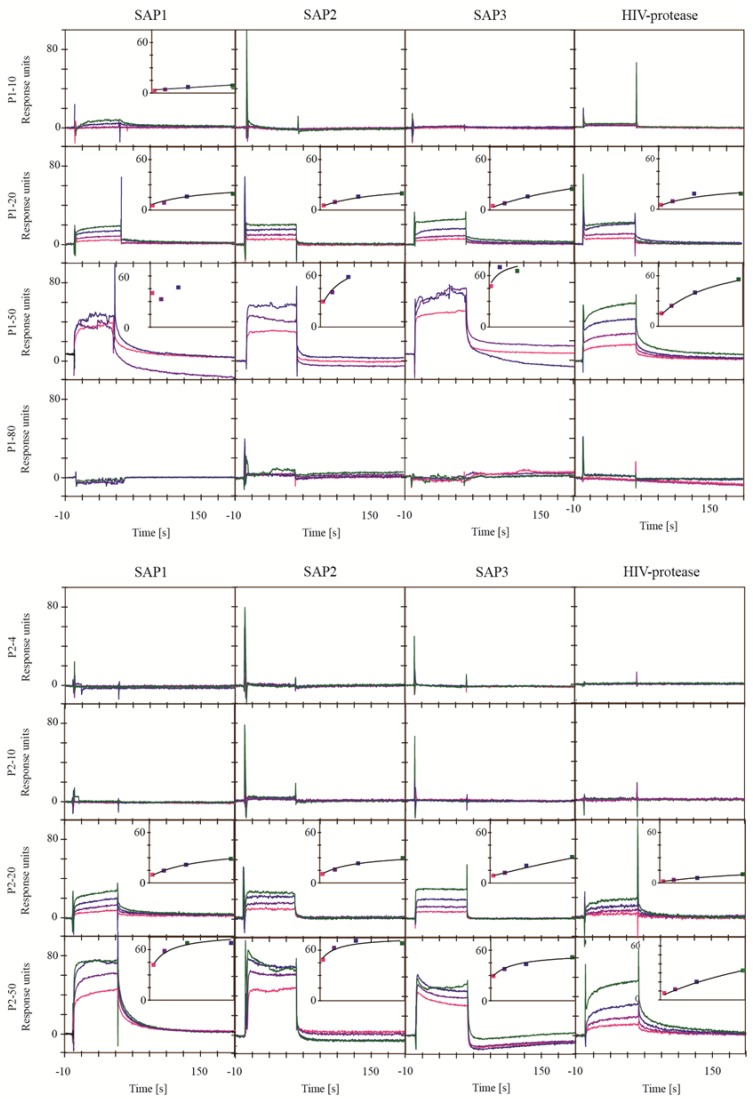
Sensorgrams from the SPR based binding assay for the interaction of the extracts with SAP1, SAP2, SAP3 and HIV-1 protease using experimental setup B. Sensorgrams for reference correction were recorded in the presence of 300 µM saquinavir for HIV-1 protease and 300 µM acetyl-pepstatin for SAP1, SAP2 and SAP3. Extracts were analyzed in dilutions of 1:80 (green), 1:160 (blue), 1:320 (purple) and 1:640 (pink). Responses are shown as absolute responses. Insets show the steady state plots.

Every extract was analyzed at four different dilutions with SAP1, SAP2, SAP3 and HIV-protease using experimental setup B (Figure 4). Extracts P1-20, P1-50, P2-20 and P2-50 were found to contain compounds interacting with the proteases. The association and dissociation of the interactions were fast and did not allow the determination of association or dissociation rate constants. Steady state plots showed a concentration dependency with saturation levels between 30 RU and 105 RU, which is reasonable for a specific interaction with a small molecule. For the SAP’s, the dilution 1:80 of extract P1-50 was removed from the sensorgrams due to problems with solubility, which is also reflected in the poor quality of the sensorgrams with higher dilution. Extracts P1-50 and P2-50 reached saturation, which is a strong indication for a specific interaction. The results show that the extracts contained compounds competing with the active site inhibitors used, and hence most likely bind to the active site of the proteases. All other extracts showed no or only weak signs of interactions. The results obtained for HIV-1 protease with experimental setup B were in accordance with the results obtained from experimental setup A.

No reliable SPR data were generated for pepsin due to high DMSO sensitivity of the enzyme, reported earlier [25]. The high DMSO sensitivity was also reflected in the high standard deviation of the inhibition values for pepsin from the FRET based activity assay.

The combination of the results from the FRET based activity assay and the SPR based binding assay allowed the identification of extracts containing promising protease inhibitors. Extracts P1-20 and P1-50 showed high inhibition in the FRET based activity assay. The SPR based binding assay demonstrated that the inhibition was most likely due to interaction with the active site of the proteases. Hence these extracts are interesting candidates for a further purification of the contained inhibitor. Extracts P2-20 and P2-50 showed clear signs of interaction in the SPR based binding assay, but only weak inhibition potency in the FRET based activity assay. For the HIV-1 protease even an increase in the monitored activity was observed. Although it is possible that an increase of the protease activity is caused by a direct interaction with an allosteric site, it is more likely caused by influencing assay conditions and thereby masking the potential influence of an inhibitor. It has been reported before that small amounts of organic solvents can increase the activity of proteases, e.g., trypsin [25]. However, despite the good results from the SPR based binding assay, the fractions P2-20 and P2-50 might not be good candidates for further inhibitor purification, since it is not clear that the observed interaction can inhibit the proteases. Extract P1-80 showed high inhibition potency in the FRET assay for SAP1, SAP2, SAP3 and pepsin. In contrast, the SPR studies showed no signs of interaction. The extract P1-80 contains mainly compounds with a hydrophobic character since it was prepared by elution with 80% acetonitrile during solid phase extraction. The FRET substrates also have a hydrophobic character. Hence, it is likely that the inhibition observed in the FRET based activity assay is a false positive, caused by interaction between the substrates and small molecules from the extract. Extracts P1-10, P2-4, P2-10 showed no inhibition in the FRET assay or any signs of interaction in the SPR based binding assay. These extracts are therefore not considered for further purification.

### 2.2. Screening for Inhibitors of BACE1

BACE1 belongs to the group of aspartic proteases. In contrast to other aspartic proteases, BACE1 is a transmembrane protein and only poorly inhibited by common aspartic protease inhibitors, e.g., acetyl-pepstatin [26]. It is therefore not surprising that the extracts showed different results in the FRET based activity assay for BACE1 compared with the other aspartic proteases used in this study. Only extract P1-20 showed a clear inhibition with 44% reduction of protease activity. All other extracts showed only weak inhibitions. The extracts were also analyzed in an SPR based binding assay with full length BACE1 embedded into a lipid membrane. The sensorgrams showed strong bulk effects and signs of nonspecific interactions, which did not allow any interpretations of the sensorgrams. Although it was possible to reduce the bulk effects by preparing a reference surface with BACE1 blocked by the high affinity active site inhibitor Om99-2 [27], the interpretation of the sensorgrams were still difficult and they showed no clear signs of a specific interaction (data not shown). BACE1 is a transmembrane protease and hence the immobilization for the SPR based binding assay was more complex compared to that for the other proteases used in this study [11]. The prepared surface did not only contain BACE1, but also an immobilized antibody and a lipid membrane. Especially the lipid membrane might cause strong nonspecific interaction since it can interact with a broad range of small molecules. Additionally, the complex structure of the surface increases the chances to have significant differences between the active and the reference surface, which complicates the reference corrections for removing signals from bulk effects and nonspecific interactions. Although interaction studies with pure compounds did not show any problems [11], the complex chemical composition of the extracts in combination with the complex structure of the SPR based binding assays may have generated these problems. Without any result from the SPR based binding assay, it is difficult to make assumption about the specificity of the inhibition. Hence, none of the extracts are considered for further purification. Furthermore, this shows a clear limitation of the SPR based binding assay. Despite the proofing of different experimental setups and the availability of a high affinity inhibitor, it was not possible to gain sensorgrams of good quality due to the complexity of the SPR based binding assay.

### 2.3. Screening for Inhibition of HCMV Protease

HCMV protease belongs to a special class of serine proteases and is an interesting drug target for antiviral therapy against HCMV, although no inhibitors are in clinical use yet [18]. The extracts were tested in a FRET based activity assay in a dilution 1:300. All extracts prepared with 100% MeOH (P1) inhibited HCMV protease by more than 40% with P1-20 and P1-50 showing the highest inhibitions of 71% and 68%, respectively. All extracts prepared with 5% MeOH (P2), except P2-50, showed inhibitions higher than 30% (Table 1).

**Figure 5:**
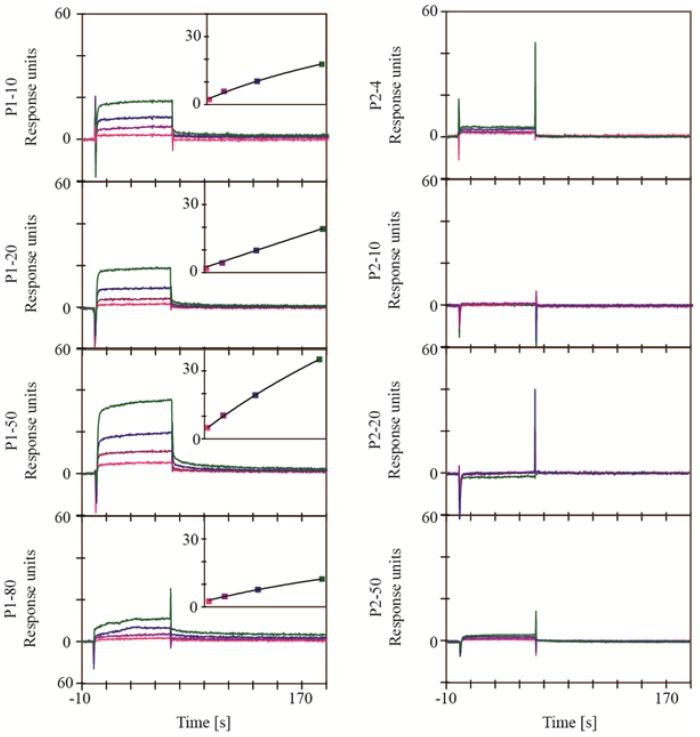
Sensorgrams from the SPR based binding assay for the interaction of the extracts with HCMV protease. Extracts were analyzed in dilutions of 1:80 (green), 1:160 (blue), 1:320 (purple) and 1:640 (pink). Responses are shown as absolute responses. Insets show the steady state plots.

In the SPR based binding assay, the extracts prepared with 100% MeOH (P1) generated sensorgrams with association and dissociation phases indicative of interacting compounds (Figure 5). Although the steady state plots showed concentration dependency, the saturation levels were as high as 3700 RU, indicating a nonspecific interaction. Since no high affinity inhibitor for HCMV protease is available, competition experiments could not be used to verify a specific interaction. This shows a limitation of the SPR based binding assay and the experimental setups used in this study, since a final confirmation of a specific interaction is dependent on the availability of a potent inhibitor. Although it cannot be completely excluded that unspecific binding masks a specific interaction, none of the extracts prepared with 100% MeOH are considered for a further purification. The extracts prepared with 5% MeOH (P2) showed only weak signs of interactions in the SPR based screening assay. This shows that the inhibition of these extracts detected in the FRET based activity assay were not caused by a specific interaction and were hence false positives.

## 3. Experimental Section

### 3.1. Preparation of Extracts from Norwegian Spring Spawning Herring

One kilogram of frozen grinded rest raw material (remaining material after fillet production) from Norwegian spring spawning herring (*Clupea harengus*) was dissolved in 4 L water and the pH adjusted to 4.5 with acetic acid. All insoluble material was separated from the solution by centrifugation for 30 min at 14,000× *g*. The supernatant was removed and a Pelicon XL/10 kDa filter was used to isolate all molecules with *M*_w_ < 10 kDa. After filtration the material was freeze dried and stored at −20 °C. The soluble material was extracted from 1 g of the freeze dried powder using four times 2 mL methanol/0.025% trifluoroacetic acid (TFA). Insoluble materials were removed by centrifugation for 5 min at 800 *g*. In a second step, the extraction was repeated with two times 2.5 mL 5% methanol/0.025% TFA. All extracts were again freeze dried and stored at −20 °C.

The freeze dried extracts were dissolved in water with 0.1% TFA and further fractionated by solid phase extraction using a RapidTrace Workstation (Caliper Life sciences, Hopkinton, MA, USA). The extracts were applied to a 200 mg Sep-Pak C18 cartridge (Waters, Milford, MA, USA), washed with 3 mL water with 0.1% TFA and eluted with different concentrations of acetonitrile (Figure 1). All extracts were analyzed by HPLC using a LC-20A prominence system (Shimadzu, Duisburg, Germany) and a SymmetrieShield RP18 column (3.5 µm, 3.0 mm × 20 mm, Waters, Milford, MA, USA). The mobile phase was composed of 2% ACN and 0.1% formic acid. During elution, the acetonitrile (ACN) concentration was increased to 98% in a linear gradient within 4 min. For the activity, assays and binding assay all samples were freeze dried and dissolved in as little DMSO as practically possible.

### 3.2. Protease Production and FRET Based Activity Assay

Proteases were recombinantly expressed and purified or purchased from commercial sources. FRET based activity assays were used to determine the influence of the extracts on the protease activity. All extracts were tested at a final dilution of 1:300 and 1:600. The substrates and the extracts dissolved in pure DMSO were diluted with buffer to match the DMSO concentration of the assay buffers. Signal increases were recorded with a fluorescence plate reader for 10, 20 or 30 min dependent on the enzyme activity. All activity measurements were done as duplicates. The mean values of the duplicates were used to calculate the percentage of enzyme inhibition by comparing the signal increases with a reference without extracts. The final percentage of enzyme inhibition was calculated as average from three independent experiments. Errors were calculated as standard deviation.

#### 3.2.1. HIV-1 Protease

The enzyme was recombinantly expressed in *Escherichia coli*, purified and the activity confirmed according to published procedures [9]. The FRET assay was carried out with the purified enzyme and an internally quenched peptide substrate DABCYL-γAbu-Ser-Gln-Asn-Tyr-Pro-Ile-Val-Gln-EDANS (Bachem, Bubendorf, Switzerland). The final concentration in each well was 15 nM HIV-1 protease and 10 µM substrate. The assay buffer consisted of 100 mM Na-acetate, 50 mM NaCl, pH 5.0 and 5% DMSO.

#### 3.2.2. SAP1, SAP2 and SAP3

SAP1, SAP2 and SAP3 from *Candida albicans* were expressed, purified and the activity tested according to published procedures [28]. The custom synthesized FRET substrate DABCYL-Lys-Pro-Phe-Glu-Leu-Phe-Lys-Leu-Glu-EDANS (Biomatik, Wilmington, DE, USA) was used at a concentration of 3.33 µM. The final enzyme concentration was 5.3 nM for SAP1, 1.6 nM for SAP2 and 31.3 nM for SAP3. The assay buffer contained 100 mM Na-acetate, 150 mM NaCl, pH 3.8 and 5% DMSO.

#### 3.2.3. Pepsin

The protease was purchased from Sigma-Aldrich (St. Louise, MO, USA) and the FRET substrate MOCAC-Ala-Pro-Ala-Lys-Phe-Phe-Arg-Leu-Lys(Dnp)-NH_2_ from Peptide (Osaka, Japan). The assay was carried out in 0.1 M formic acid buffer, pH 3.0 with an enzyme concentration of 1.1 nM and a final substrate concentration of 1.6 µM.

#### 3.2.4. BACE1

Full length BACE1 was expressed in Sf9 cells. For the FRET based activity assay, the Sf9 cells were lysed in PBS with 2% Triton and all insoluble material was removed by centrifugation. The supernatant was directly added to the internally quenched substrate EDANS-Glu-Val-Asn-Leu-Asp-Ala-Glu-Phe-Lys-DABCYL (Bachem, Bubendorf, Switzerland) at a final substrate concentration of 4.9 µM in buffer consisting of 100 mM Na-acetate, 50 mM NaCl, pH 4.5, 5% DMSO and 2% Triton. The FRET assay and the protein expression were carried out as previously described [11].

#### 3.2.5. HCMV Protease

The enzyme was expressed in Escherichia coli and purified according to published procedures [29,30]. The internally quenched peptide DABCYL-Arg-Gly-Val-Val-Asn-Ala-Ser-Ser-Arg-Leu-Ala-EDANS (Bachem, Bubendorf, Switzerland) was used as FRET substrate at a final concentration of 1.25 µM. The final enzyme concentration was 33 nM. The assay buffer contained 100 mM TES, 50 mM NaCl pH 7.6, 0.1 mM EDTA 15% glycerol and 5%DMSO.

### 3.3. SPR Based Binding Assays

All SPR assays were performed at 25 °C with Biacore S51 or Biacore 2000 instruments (GE Healthcare, Uppsala, Sweden). The extracts were injected for 60 s at dilutions of 1:80, 1:160, 1:320 and 1:640. The dissociations were recorded for 2 min.

#### 3.3.1. HIV-1 Protease

Between 3500 and 5500 RU HIV-protease was immobilized and cross linked as previously described [9]. All experiments were carried out in 100 mM Hepes pH 7.4, 50 mM NaCl and 5% DMSO. The extracts were tested in two different experimental setups. In experimental setup A, reference correction was done by a surface with immobilized HIV-1 protease, where the active sites were blocked by three injections for 30 s of 1 µM saquinavir (Sigma-Aldrich, St. Louise, MO, USA) previously to every dilution series. In the experimental setup B, the sensorgrams were also recorded in the presence of 300 µM saquinavir (Sigma-Aldrich, St. Louise, MO, USA), reference corrected and subtracted from sensorgrams recorded in the absence of saquinavir.

#### 3.3.2. SAP1, SAP2 and SAP3

All SAP’s were biotinylated and immobilized as earlier described [10,28]. Shortly, the protease buffer was changed to 0.1 M phosphate buffer pH 7.2 and incubated in 1:1 molar ratio with Biotin-X-NHS (Calbiochem, San Diego, CA, USA) for 30 min at 22 °C. The final concentration of enzyme was around 2 µM. Unreacted biotin-X-NHS was removed by centrifugal filter devices with a molecular cut off ~30 kDa and the buffer changed to 100 mM Na-acetate, 150 mM NaCl and pH 4.75. For immobilization, the proteins were injected for 20 min over a surface with immobilized streptavidin (Sigma-Aldrich, St. Louise, MO, USA). The immobilization of streptavidin was carried out by standard amine coupling. The protein was dissolved in 10 mM Na-acetate pH 5.0 at a concentration of 300 µg/mL and injected for 20 min. The interaction studies with the extracts were carried out in 100 mM Na-acetate, 150 mM NaCl, pH 3.8, 0.05% Tween 20 and 3% DMSO. All extracts were analyzed in the presence of 300 µM acetyl-pepstatin (Calbiochem, San Diego, CA, USA) and the sensorgrams subtracted from sensorgrams recorded in the absence of acetyl-pepstatin. All sensorgrams were reference corrected by a surface with immobilized streptavidin.

#### 3.3.3. BACE1

Full length BACE1 was immobilized as described earlier [11]. For reference correction either a surface without BACE1 or a surface with BACE1 where the active site was blocked by three injection of 1 µM OM99-2 (Sigma-Aldrich, St. Louise, MO, USA) was used. All experiments were carried out in 100 mM Na-acetate pH 4.5, 50 mM NaCl and 5% DMSO.

#### 3.3.4. HCMV Protease

The enzyme was immobilized by standard amine coupling and cross linked [29]. The experiments were carried out in 100 mM Hepes, 50 mM NaCl, pH 7.4, 0.05% Tween 20 and 3% DMSO.

## 4. Conclusions

In this study, we showed that the combination of an activity assay and an SPR based binding assay is a powerful tool for screening marine extracts for protease inhibitors, since it allows the identification of false positive hits. Extracts from Norwegian spring spawning herring containing specific inhibitors for HIV-1 protease, SAP1, SAP2 and SAP3 were identified, which demonstrates that marine vertebrates offer an interesting source for marine drug discovery. The novel approach used in this study to screen for protease inhibitors can be easily adapted to other types of enzymes and has hence a high potential for improving marine drug discovery. Furthermore, the approach can also be used for bioactivity guided isolation of bioactive compounds.
